# Supplementary figures and images for: An association analysis between hypertension, dementia, and depression and the phases of pre-sarcopenia to sarcopenia: A cross-sectional analysis
Source: PLoS One. 2021 Jul 22;16(7):e0252784. doi: 10.1371/journal.pone.0252784 (PMC8297796; doi:10.1371/journal.pone.0252784)

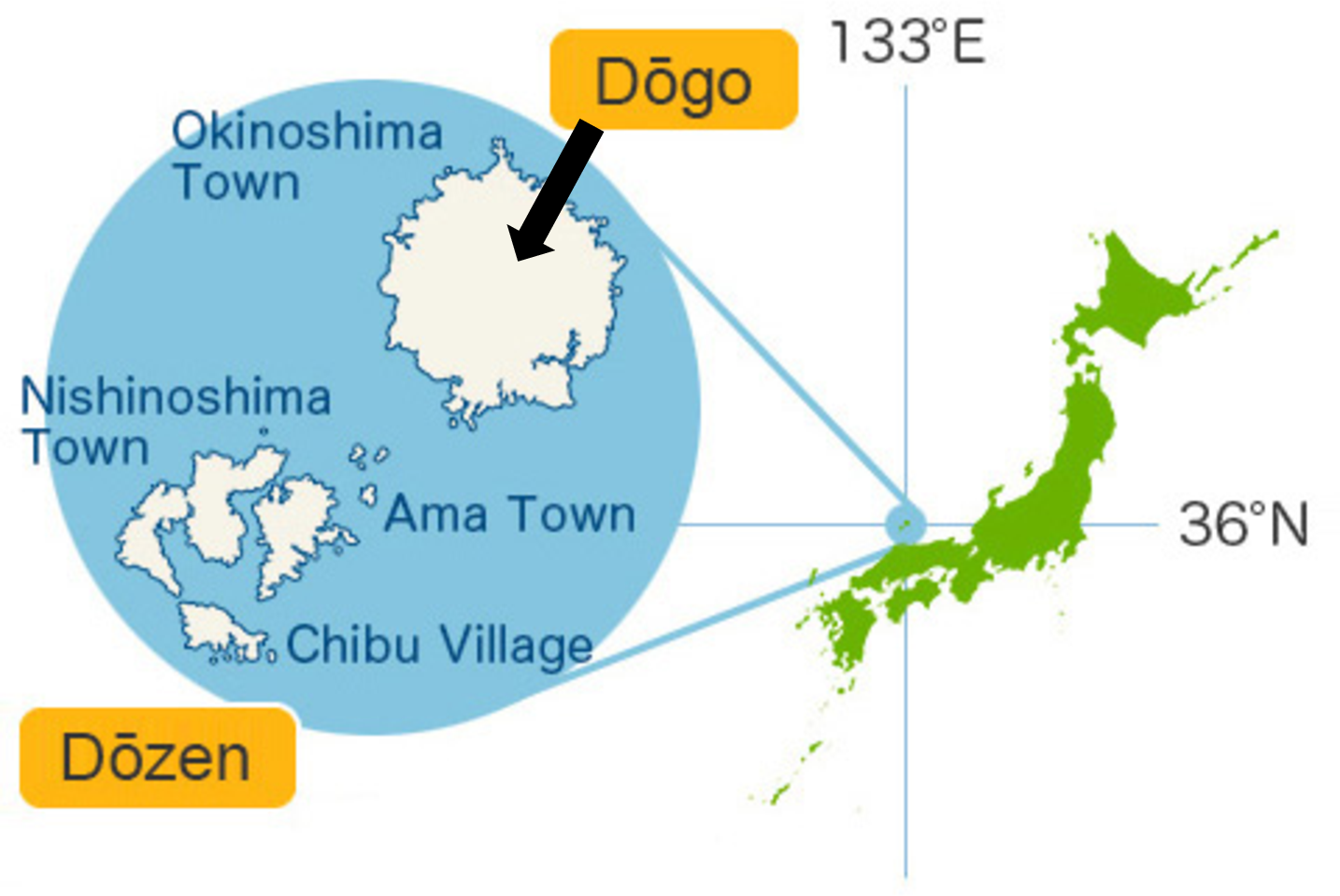

Supplement: S1 Fig — Published with permission from Oki Islands UNESCO Global Geopark Promotion Committee. Available from: http://www.oki-geopark.jp/en/features/ (accessed July 22, 2020). (TIF) [file pone.0252784.s001.tif]

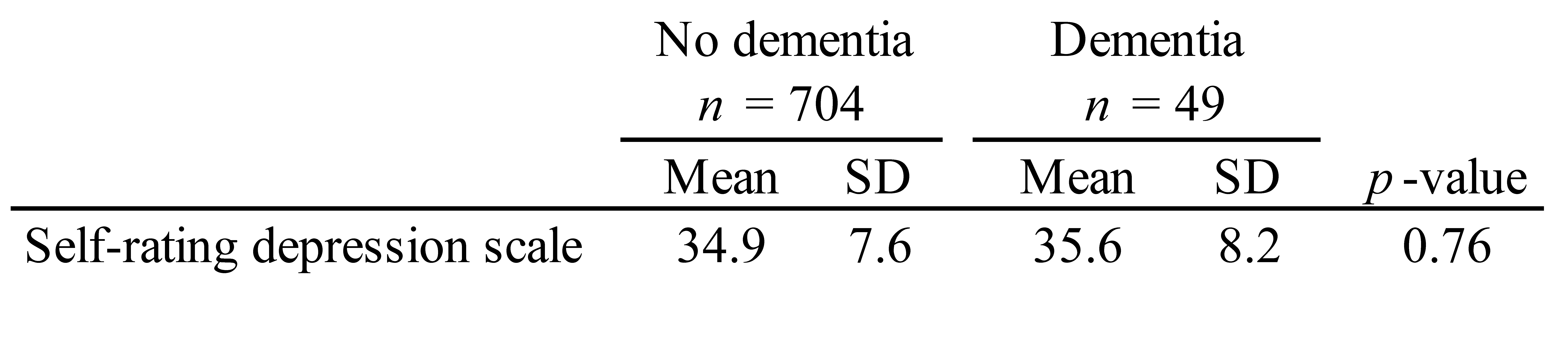

Supplement: S1 Table — (TIFF) [file pone.0252784.s002.tiff]

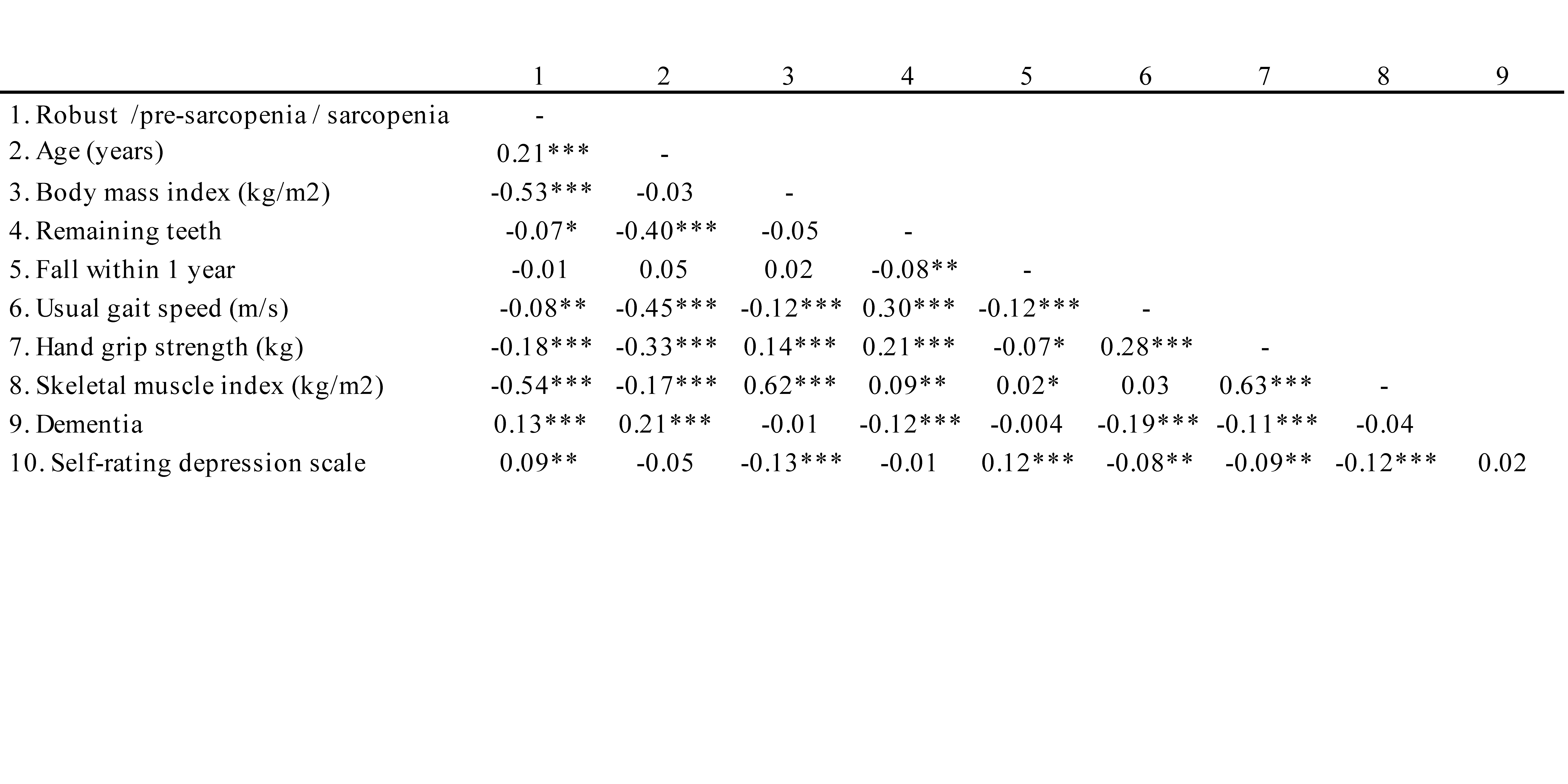

Supplement: S2 Table — * p < 0.10,** p < 0.05, *** p < 0.01. (TIFF) [file pone.0252784.s003.tiff]

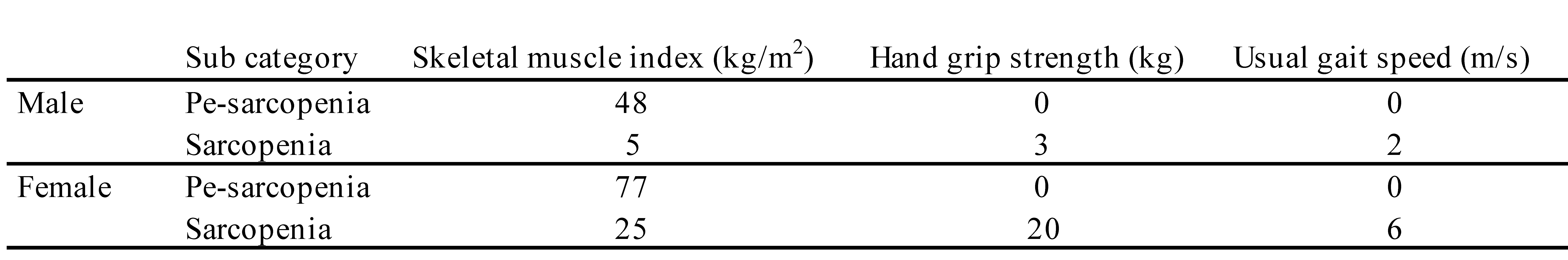

Supplement: S3 Table — (TIFF) [file pone.0252784.s004.tiff]
