# Supplementary material for: An association analysis between hypertension, dementia, and depression and the phases of pre-sarcopenia to sarcopenia: A cross-sectional analysis
Source: PLoS One. 2021 Jul 22;16(7):e0252784. doi: 10.1371/journal.pone.0252784 (PMC8297796; doi:10.1371/journal.pone.0252784)
Supplement: Attachment — Submitted filename: PLOSONE 20201208.docx [file pone.0252784.s006.docx]

I found the study titled " The transition phase of pre-sarcopenia to sarcopenia association analysis between hypertension, dementia, and depression: A cross-sectional study" addresses an interesting topic on spectrum of sarcopenia in community-dwelling older adults. However, I noted that there are tremendous rooms for improvement for the manuscript to provide enough explanation that the data deserves, and to add some clinical knowledge in the field of geriatrics.

Major points>

1) Throughout the manuscript: By using the term ‘transition’, authors may mislead readers on the nature of the study, since this cross-sectional study only compares clinical factors across non-sarcopenic, pre-sarcopenic, and sarcopenic participants in this community-based study while the term transition usually denotes some longitudinal dynamic changes of a condition.

2) Introduction: This part should be improved to provide rational backgrounds and needs to support the conceptualization of the study hypothesis. Irrelevant remarks in the introduction part (e.g., line 57-58, Cognitive decline due to…) should be removed. In the last part, study hypothesis should be more clearly addressed. Also, on top of previous researches that authors remarked in the last part of the introduction, I hope to see what the current study adds up as a meaningful scientific literature.

3) Analysis/results: While authors used logistic regression analysis, I think factors associated with sarcopenia spectrum can be more clearly assessed using ANCOVA, with no-sarcopenia, presarcopenia, and sarcopenia as independent variables. Maybe as a sensitive analysis. As a recent paper suggested (Jang et al, JCSM 2020 Apr; 11(2): 497–504), spectrums of sarcopenia might be incremental and somehow continuous, highly correlate with frailty spectrum. Therefore, performing multiple comparisons between 3 conditions of no sarcopenia, pre-sarcopenia, and sarcopenia might be less meaningful.

4) Discussion, Line 288: In the current study design, mechanism cannot be drawn. These remarks seem to be rather speculative.

4) Discussion, Line 307: Irrelevant (or less relevant) remarks for current study and analysis.

5) Conclusion: Conclusion should concisely summarize the main hypothesis and corresponding study results, and interpretations from authors.

Minor points>

1) Co-first authors are not marked in the author list

2) I recommend authors rather not to use the term elderly in the scientific literature, (See Vaughan et al, JAGS, 2019 67:211-217), and replace it with case-specific terms such as older people, older participants, individuals etc. While authors are using the term ‘subject’, I also noted that this word can be replaced with ‘participants’ in the present study is an observational study. In choosing words describing sex/gender, I recommend to have some unity, for example sex/male/female vs. gender/men/women

3) Page 8 Line 131, Is the term physique a commonly used word in scientific literature?

4) Table 1, n=753 is everywhere, except for fall item (n=735). I recommend authors to provide total n (753) in general), and provide n of fall item in the note with appropriate quotation marks for the journal. P values should be provided with 3 digits below decimal points. I also opposite in separating table into 2 vertical sections of mean+-sd and n(%), but this might be up to the journal’s policy.
