# Supplementary material for: An association analysis between hypertension, dementia, and depression and the phases of pre-sarcopenia to sarcopenia: A cross-sectional analysis
Source: PLoS One. 2021 Jul 22;16(7):e0252784. doi: 10.1371/journal.pone.0252784 (PMC8297796; doi:10.1371/journal.pone.0252784)
Supplement: Attachment — Submitted filename: Respons to Reviewers.docx [file pone.0252784.s007.docx]

**Response to Reviewer 1**

Thank you for pointing out the most fundamental part of the definition and classification of sarcopenia. Please also note that we have included a description of why we did not follow the AWGS 2019 criteria.

1. Introduction: 1. The authors stated that "sarcopenia is a phenotype of frailty". This statement is not based on evidence. It was just a consideration from some researchers. Some authors argued that frailty and sarcopenia were "two sides of one coin." Please try to use the widely agreement of the definition of sarcopenia, such as EWGSOP2 or AWGS.

**Reply:** As you pointed out, the description seemed to confuse sarcopenia with frailty, so we changed it to something widely agreed upon.

**Revised: Page 5, line 62-63 <Introduction>**

Sarcopenia is a geriatric syndrome, characterized by age-related weakness of skeletal muscles as well as loss of muscle strength and physical function

2. Introduction: 'Sarcopenia can be classified into three stages: robust, pre-sarcopenia, and sarcopenia'. This statement is not true. Robust is not a stage of sarcopenia. Sarcopenia can be classified into three stages: pre-sarcopenia, sarcopenia, and severe sarcopenia.

**Reply:** Thank you for pointing out this error. We had to differentiate between older people’s medical condition and not sarcopenia. We have corrected it as follows.

We did not include severe sarcopenia because the purpose of this study was to analyze the characteristics of pre-sarcopenia. Furthermore, the study was conducted in 2016, and we followed the criteria of AGWS 2014 and not AGWS 2019, which incorporated severe sarcopenia. Please see the response to comments 6 and 8 for a more detailed explanation.

**Revised: Page 5, line 76-77**

Older people can be classified into three stages: robust, pre-sarcopenia (i.e., decrease in muscle mass, but not in gait speed or grip strength), and sarcopenia.
3. Figure 1 is not the main results of this paper. It should serve as a supplementary figure, if necessary.

**Reply:** I followed your instructions and changed it to a supplementary file.

4. This a secondary analysis of a cross-sectional study. This point should be clearly stated.

**Reply:** This is the first analysis conducted by a cross-sectional study. The authors of this paper, who belonged to CoHRE, planned, acquired data, and created the database. This part was not written properly, so I added it to the Method section.

**Revised:** Page **7-8, line 116-118**5.

5. We need more details about the handgrip strength measurement and walking speed measurement.

**Reply:** Thank you for pointing this out. I added a note about the measurement method and its definition of decline.

**Revised: Page 10-11, Line 166-172 <Method>**

Handgrip strength of each hand was measured twice by using a grip strength dynamometer (Takei Scientific Instruments: Niigata, Japan). Data were collected based on maximum grip strength in kilograms (kg) to the first decimal place. Usual gait speed was measured twice by trained examiners using digital stopwatches on a 5 meter course set between 3- and 8-meter marks on an 11 meter straight, flat, indoor walkway. We asked participants to walk as usual on the walkway course. Results were recorded in seconds to the first decimal place.

6. The authors defined sarcopenia based on the AWGS 2014. Why didn't they use the AWGS 2019? Would the results significantly change if the AWGS 2019 definition of sarcopenia was applied? Perhaps, the authors can also use the AWGS 2019 criteria and reanalysis their data to perform a sensitivity analysis of their results in order to test the robustness of their conclusion.

**Reply:** We believe that in remote areas with few medical resources, prevention of sarcopenia is an important issue, and this study focused on extracting the characteristics of pre-sarcopenia. Since the purpose of this study was not properly communicated, I have included the purpose in the last sentence of the introduction so that the purpose is clear again.

The sarcopenia study you mentioned, using the AWGS 2019 criteria, is our next research study. CoHRE is still conducting annual surveys in the same way, and a paper on sarcopenia using the AWGS2019 criteria is being prepared using data from Oki Island.

**Revised: Page 7, Line 98-101 <Introduction>**

The purpose of this study is to examine the variables that influence the shifting phase of sarcopenia in the residents of Oki-island. We hypothesized that depression and dementia have a positive effect, while hypertension has a negative effect, on the transition process of sarcopenia.

7. "Chronic diseases (hypertension, dyslipidemia, diabetes, cardio-cerebrovascular disease; no = 0, yes = 1)" What about other important diseases such as COPD, liver disease, and CKD?

**Reply:** We did not obtain the data that you mentioned at the time of our 2016 survey. We are currently acquiring data on liver diseases and will consider acquiring data on COPD and CKD in the future.

8. Why did the authors not define severe sarcopenia since they have the data on ASMI, handgrip strength, and walking speed?

**Reply:**

This study was conducted in 2016, and the speed of the 5m gait was measured according to the AWGS 2014 criteria, not the 6m gait used in AWGS 2019. Other methods of case selection such as lower leg circumference and SARC-F recommended by AWGS 2019 were not assessed. Therefore, it is difficult to reanalyze the data using the criteria of AWGS 2019 because the inclusion criteria was different. As a trial, we tried to diagnose severe sarcopenia using the AWGS 2019 criteria using SMI, walking speed, and hand grip strength, but the number of participants was as low as nine. The participants were community-dwelling health screening participants, who had a higher percentage of pre-sarcopenia than sarcopenia. As we noted in reply to comment 6, a sarcopenia study using the AWGS 2019 criteria is our future research.

9. Did the authors consider the multicolinearity in their models?

**Reply:** First, the correlation coefficients between each variable were calculated, and adjustments were made so that variables that were strongly correlated, such as BMI and SMI, were not entered simultaneously. Further, both the ordered logistic regression and the variance inflation factor of the variables found in the multiple logistic regression analysis were less than 10. This was added to the results.

**Revised: Page 18, Line 272-274 <Result>**

The variance inflation factors of the variables used in the multivariate analysis conducted in this study were less than 10.

10. The authors reported that “all the continuous variables were not normally distributed” . However, they reported mean and SD for these variables in Table 1.

**Reply:** I created a new table 1 with changes to median and inter quartile range.

11. The prevalence of sarcopenia is extremely low (3.9%) compared to the data in the literature. Is there any explanation for these results? Maybe there are selection bias as the participants were recruited from group health examination. As a result, the generalizability of this paper would be weakened.

**Reply:** As mentioned in Page19, line 278-281, this study was based on health examination data of relatively active older people who were able to come voluntarily to the study site, and the incidence of sarcopenia was low. The main purpose of this study was to pick up relevant factors in participants with pre-sarcopenia, and we believe that the design was able to fulfill that purpose. However, as you pointed out, the findings of this study do not apply to populations with a high prevalence of sarcopenia, such as community-dwelling older people, including those who are bedridden in home care or institutionalized patients. This was not explained well enough, so I added this information in the discussion section.

**Revised:　Page 24, Line 333-337 <Discussion>**

The sarcopenia rate in our study was 3.9 %, lower than in a previous study which reported a rate of approximately 10.0 % [52, 53]. This may be because the participants in our study were active enough to participate in a voluntary health checkup, whereas bedridden and institutionalized residents could not participate in the checkups.

**Response to Reviewer 2**

We greatly appreciate your comments. I learned that sarcopenia is some kind of continuous state change and have revised our manuscript accordingly.

Major points>
1) Throughout the manuscript: By using the term ‘transition’, authors may mislead readers on the nature of the study, since this cross-sectional study only compares clinical factors across non-sarcopenic, pre-sarcopenic, and sarcopenic participants in this community-based study while the term transition usually denotes some longitudinal dynamic changes of a condition.
**Reply:** As you pointed out, we realized that using "transition" in our research results misleads people into thinking that it is a longitudinal study. We deleted the word “transition” and changed it to "shift”.

2) Introduction: This part should be improved to provide rational backgrounds and needs to support the conceptualization of the study hypothesis. Irrelevant remarks in the introduction part (e.g., line 57-58, Cognitive decline due to…) should be removed. In the last part, study hypothesis should be more clearly addressed.

**Reply:** The parts you have highlighted were not clearly related to the main discussion; hence, I have deleted the text and made revisions accordingly.

**Revised: Page 7, Line 98-101 <Introduction>**

We hypothesized that depression and dementia have a positive effect, while hypertension has a negative effect, on the transition process of sarcopenia.

2) Also, on top of previous researches that authors remarked in the last part of the introduction, I hope to see what the current study adds up as a meaningful scientific literature.
**Reply:** We believe that prevention of sarcopenia in remote areas where medical resources are scarce is important, and our main focus was to extract the characteristics of pre-sarcopenia. Since we did not explain this purpose properly, we stated it in the last sentence of the introduction to clearly convey the purpose.

**Revised: Page 7, Line 98-99**

The purpose of this study is to examine the variables that influence the shifting phase of sarcopenia in the residents of Oki-island.

3) Analysis/results: While authors used logistic regression analysis, I think factors associated with sarcopenia spectrum can be more clearly assessed using ANCOVA, with no-sarcopenia, presarcopenia, and sarcopenia as independent variables. Maybe as a sensitive analysis. As a recent paper suggested (Jang et al, JCSM 2020 Apr; 11(2): 497–504), spectrums of sarcopenia might be incremental and somehow continuous, highly correlate with frailty spectrum. Therefore, performing multiple comparisons between 3 conditions of no sarcopenia, pre-sarcopenia, and sarcopenia might be less meaningful.

**Reply:** Thank you for suggesting a better analysis. The purpose of this study is to analyze the process of transition to sarcopenia. We are in complete agreement that spectrum of sarcopenia is some kind of a continuous. Therefore, we also believe that sarcopenia is a gradual and sequential transition, such as robust, pre-sarcopenia, and sarcopenia, and we conducted ordered logistic regression, a logit analysis that can track the sequential transition. As you pointed out, ANCOVA may be more appropriate if these three are considered continuous. ANCOVA with robust, pre-sarcopenia, and sarcopenia assigned as dependent variables (0, 1, and 2) showed a significant association between age and diabetes mellitus (additional file). Thank you for your guidance on other analysis methods. However, we would like to leave this as a future issue because looking at the correlation between sarcopenia and frailty spectrum with this kind of method is far from the main purpose of this study.

In our introduction and discussion sections, I have added the findings that sarcopenia is some kind of continuous state that correlates with frailty index (Jang et al, JCSM 2020 Apr; 11(2): 497–504). I also learned that the frailty index used here has been studied in the Asian region for a long time and is an index that can be applied to Japanese people without problems (Jung et al, PLoS One. 2014 Feb 4;9(2):e87958). I have also added this finding to the text.

This study is consistent with the purpose of our research, which showed that people with pre-sarcopenia, who are not considered to have sarcopenia because their grip strength is preserved, also have a risk for adverse outcomes. Also, the purpose of comparing these three states and the hypothesis is more clearly stated in the last paragraph of the introduction.

**Revised: Page 5, Line 52-54 and 63-65<Introduction>**

Jang et al. demonstrated the possibility that spectrums of sarcopenia may be in a continuous state of change that correlates with measures of frailty [2, 3].

**Page 5-6, Line 77-79 <introduction>**

Previous studies have shown that people who are not considered to have sarcopenia because of preserved grip strength also have an increased risk for adverse outcomes [2].

**Page 23, Line 309-312**

These results indicated that a steady decline in individuals’ mental and physical status, led to the development of sarcopenia; moreover, this finding is consistent with a previous study, indicating that the shift from robust to sarcopenia is a continuous state of change that correlates with frailty [2].

4) Discussion, Line 288: In the current study design, mechanism cannot be drawn. These remarks seem to be rather speculative.
**Reply:** You pointed out the part where you speculate why hypertension was less in pre-sarcopenia patients. We have added a previous study on the relationship between hypertension and pre-sarcopenia and have stated that our results are consistent with them and made some assumptions about the mechanisms involved.

**Revised: Page 25, Line 352-357<Discussion>**

In this study, hypertension was significantly less common in pre-sarcopenia patients than in robust patients. This was consistent with a previous study [31]. In general, nutritional therapy and exercise therapy are recommended for the management of hypertension [57], and these two therapies have been reported to be effective in preventing sarcopenia [4, 5]. However, the relationship between hypertension and prevention of sarcopenia requires further research.

5) Discussion, Line 307: Irrelevant (or less relevant) remarks for current study and analysis.

**Reply:** We have confirmed that the content was indeed irrelevant to the text. Therefore, I have deleted the text.

6) Conclusion: Conclusion should concisely summarize the main hypothesis and corresponding study results, and interpretations from authors.

**Reply:** I clarified the description of the hypothesis in the introduction, and based on that, I revised the conclusion per your suggestion.

**Revised: Page 27, Line 382-385 <Conclusion>**

　　Depression was positively associated to the shifting phase from robust to pre-sarcopenia, while hypertension was negatively associated; however, dementia had no significant effect. The depression scale of the rural island residents needs to be followed up because it can be an associated factor in the future progression of sarcopenia.

Minor points>
1) Co-first authors are not marked in the author list
Reply: The co-first authors are now marked on the title page.

**Revised: Page 1, Line 5-6**

Takeshi Endo^1,2*¶^, Tsunetaka Kijima^2,3¶^, Shigetaka Kitahara^4&^, Takafumi Abe^2&^, Miwako Takeda^2&^, Shozo Yano^2,5&^, Minoru Isomura^2,6&^, Toru Nabika^2,7&^ and Kenju Akai^2¶^

2) I recommend authors rather not to use the term elderly in the scientific literature, (See Vaughan et al, JAGS, 2019 67:211-217), and replace it with case-specific terms such as older people, older participants, individuals etc. While authors are using the term ‘subject’, I also noted that this word can be replaced with ‘participants’ in the present study is an observational study. In choosing words describing sex/gender, I recommend to have some unity, for example sex/male/female vs. gender/men/women
**Reply:** We have unified the terminology as you suggested.

**Revised: Throughout the entire manuscript.**

3) Page 8 Line 131, Is the term physique a commonly used word in scientific literature?
**Reply:** The term "physique" has been replaced by "anthropometry" because of a misinterpretation of the terminology.

**Revised: Page 10, Line 155 <Method>**

**Measurement of anthropometry and skeletal muscle mass**

4) Table 1, n=753 is everywhere, except for fall item (n=735). I recommend authors to provide total n (753) in general), and provide n of fall item in the note with appropriate quotation marks for the journal. P values should be provided with 3 digits below decimal points. I also opposite in separating table into 2 vertical sections of mean+-sd and n(%), but this might be up to the journal’s policy.

**Reply:** We have revised Table 1 accordingly.

**Revised: Page 15, Line 249 <Table 1.>**

**Response to Reviewer 3**

We greatly appreciate your specific guidance on what needs to improve in terms of BMI numbers, incidence of sarcopenia, and AGWS criteria.

Major comments:
1. The authors described traditional risk factors for sarcopenia and the current state of the rural island in the introduction. However, the purpose of the study was unclear, so please include this at the end of the introduction.

**Reply:** As you pointed out, the part describing the purpose of the research was unclear; hence, I have added it to the last paragraph of the introduction.

**Revised: Page 4, Line 52-54 and 65-66<Introduction>**

The purpose of this study is to examine the variables that influence the shifting phase of sarcopenia in the residents of Oki-island.

2. The presence or absence of obesity (≥25 or 30 kg/m2) is also an important independent factor for sarcopenia; however, why did you mention hypertension, diabetes, and cerebrovascular disease without including obesity?
**Reply: As you pointed out,** BMI is an important index that is also related to muscle mass and mortality [Abramowitz MK, 2018;13(4):e0194697. Epub 2018/04/12], and obesity has been reported to be a risk factor for sarcopenia [Batsis JA, Nat Rev Endocrinol. 2018;14(9):513-37]. BMI was not included as a covariate because skeletal muscle index, one of the diagnostic criteria for sarcopenia, had a very high correlation with BMI. Including BMI in the ordered logistic regression with Robust/Pre-sarcopenia/Sarcopenia as the dependent variable would result in high multicollinearity. However, obesity is a very important index for sarcopenia, and we have added it to the results.

**Revised: Page 15, Line 240-241<Result>**

Among the participants in this study, 23.6% had BMI ≥ 25 kg/m^2^ and 2.5% had BMI ≥ 30 kg/m^2^.

3. The comparison of the three groups was performed using the Kruskal-Wallis test or χ2 test, but no ad hoc analysis was performed, such as multiple comparison or residual analysis.
**Reply:** The purpose of our study is to show a stepwise trend and not to look at the difference between two groups in an ad hoc multiple comparison. For this reason, we used ordered logistic regression as our method. I explicitly added the reason for using this method in the analysis section.

Reviewer 2 also suggested that we use ANCOVA, and we explained accordingly.

4. The prevalence of sarcopenia in this study was 3.9%, which is very low compared with those reported in other studies. It is necessary to explain the reason and characteristics of the low prevalence of sarcopenia, including the study of rural islands.
**Reply:** I searched the previous study of sarcopenia prevalence in remote island in Japan and the prevalence was also higher than our study. However we checked the details of how the participants were recruited in the previous study, but there was no mention of whether bedridden patients or other physically weakened people. We thought that the lower prevalence of sarcopenia in our study than in previous studies was due to the fact that the participants were relatively active older people who were able to voluntarily come to the study site. I have revised as follows.

**Revised: Page 24, Line 333-337**

The sarcopenia rate in our study was 3.9 %, lower than in a previous study which reported a rate of approximately 10.0 % [52, 53]. This may be because the participants in our study were active enough to participate in a voluntary health checkup, whereas bedridden and institutionalized residents could not participate in the checkups.

5. There were sex-based differences in this study. Please clarify why you did not analyze by sex.

**Reply:** As you pointed out, sex was a significantly related variable between the pre-sarcopenia and sarcopenia groups (Table 3.3). However, in the characteristics table (Table 1) and the ordered logistic regression table (Table 2), and multiple logistic regression (Table 3.1, 3.2) sex was not a significantly associated factor. Since the main purpose of this study was to extract the characteristics of pre-sarcopenia and other stages, we did not conduct the analysis with sex as the main axis. The difference between pre-sarcopenia and sarcopenia is not in muscle mass but in muscle strength, that’s why sex may be related to this part. I also made a comparison table of SMI, hand grip strength, and usual gait speed of the two groups and added it to the results as an additional file (S3 table). The reason for the association between women and sarcopenia is that there were more women with sarcopenia who had low hand grip strength. Thank you for presenting a new perspective. I have added the following to the text.

**Revised: Page 22, Line 302-304**

S3 Table shows the number of participants who fell below the diagnostic criteria for sarcopenia was divided by gender and sub-category (pre-sarcopenia and sarcopenia).

**Revised: Page 25-26, Line 358-367<Discussion>**

In this study, there were significantly more females in the sarcopenia group than in the pre-sarcopenia group. The two groups are defined by the difference in muscle strength in which pre-sarcopenia has preserved muscle strength while sarcopenia has decreased muscle strength. According to the AWGS 2014 criteria for sarcopenia, this muscle strength is defined as SMI (<5.7 kg/m2 for female and <7.0 kg/m2 for male) and grip strength (<18 kg for female and <26 kg for male), with a lower cutoff point for women. In the sarcopenia group of this study, it was clear that there were more females with grip weakness than male (S3 Table). Further additional studies are needed to clarify the causal relationship between gender and this muscle weakness.

Minor comments:
1. It is recommended that the island description and figure 1 provided in the introduction are presented in the participants section of Materials and Methods.
**Reply:** I changed the location of figure 1 as you instructed. Also, as pointed out by another reviewer, I made this figure an additional file because this figure is not what we have clarified in this study.

2. The current diagnosis of sarcopenia generally uses AWGS2019; however, it is important to explain why AWGS was used.
**Reply:** We believe that in remote areas with few medical resources prevention of sarcopenia is an important issue, and this study focused on extracting the characteristics of pre-sarcopenia. Since the purpose of this study was not properly communicated, I have included the purpose and hypothesis in the last sentence of the introduction. Also, this study was conducted in 2016, and the speed of the 5m walk was measured according to the AWGS 2014 criteria, not the 6m walk used in AWGS 2019. Other methods of case selection such as lower leg circumference and SARC-F recommended by AWGS 2019 were not assessed. Therefore, it is difficult to reanalyze the data using the criteria of AWGS 2019 because the inclusion criteria was different. As a trial, we tried to diagnose severe sarcopenia using the AWGS 2019 criteria using SMI, walking speed, and hand grip strength, but the number of participants was as low as nine. The participants were community-dwelling health screening participants, who had a higher percentage of pre-sarcopenia than sarcopenia. CoHRE is still conducting annual surveys in the same way, and a paper on sarcopenia using the AWGS 2019 criteria is being prepared using data from Oki Island. I have added a note to Limitation about why we could not use the AWGS2019 criteria.

**Revised: Page 6, Line 95-96　<Introduction>**

The purpose of this study is to examine the variables that influence the shifting phase of sarcopenia in the residents of Oki-island. We hypothesized that depression and dementia have a positive effect, while hypertension has a negative effect, on the transition process of sarcopenia.

**Revised: Page Page 26, Line 374-378 <Limitaion>**

Third, this study was conducted in 2016, and the speed of the 5 m gait was measured according to the AWGS 2014 criteria, and not the AWGS 2019 criteria using the 6 m gait. Thus, other methods of case selection such as lower leg circumference and SARC-F, recommended by AWGS 2019, were not assessed. Our future task is to a conduct a sarcopenia study using the AWGS 2019 criteria.

3. Please add the definition or cutoff for dementia diagnosis by CADi2 in the “screening for dementia” section.
**Reply:** As you pointed out, the cut off was unclear. This cognitive function test is somewhat complicated, as the test takes into account not only the score but also the response time. I have added the information to the Method in the text.

**Revised: Page 12, Line 194-199, <Method>**

The measured CADi2 score was considered as cognitive decline if the cutoff score was 6 points or less. In addition, participants who were 74 years old or younger and had a response time of 200 seconds or longer or were 75 years old or older and had a response time of 250 seconds or longer, were considered to have cognitive decline regardless of their CADi2 score. In this study, participants who were considered to have such cognitive decline were defined as the dementia group.

4. The non-normal distribution data in Table 1 should be presented as median and interquartile range.

**Reply:** I should have done exactly that. I followed your instructions and created a new table 1 with changes to median and inter quartile range.

5. The following research showed that hypertension was a protective factor for sarcopenia. Hence, I recommend that you refer to it as supporting evidence:
Kurose S et al, Sci Rep 10:19129, 2020.

Reply: Thank you for the information. We have checked the contents and found them to be very important for our research. I have added it to the introduction and discussion.

**Revised: Page 6-7, Line 95-97 <Introduction>**

Hypertension has been reported to reduce the risk of sarcopenia [31] and is considered an important variable that needs further analysis.

**Revised: Page 25, Line 352-357<Discussion>**

In this study, hypertension was significantly less common in pre-sarcopenia patients than in robust patients. This was consistent with a previous study [31]. In general, nutritional therapy and exercise therapy are recommended for the management of hypertension[57], and these two therapies have been reported to be effective in preventing sarcopenia[4, 5]. However, the relationship between hypertension and prevention of sarcopenia requires further research.
