# Supplementary material for: An association analysis between hypertension, dementia, and depression and the phases of pre-sarcopenia to sarcopenia: A cross-sectional analysis
Source: PLoS One. 2021 Jul 22;16(7):e0252784. doi: 10.1371/journal.pone.0252784 (PMC8297796; doi:10.1371/journal.pone.0252784)
Supplement: Attachment — Submitted filename: Response_to_reviewers.docx [file pone.0252784.s008.docx]

Response to reviewer 3

Reviewer 3

Major comments:
1. The expression “shifting phase” is inappropriate in this study. Because the authors have not examined the predictors of subject who have shifted from non-sarcopenia to pre-sarcopenia. This study is a cross-sectional study, and its influence on the shifting phase is unclear.

**Reply:**

　I changed the word to shifting instead of transition as pointed out by another reviewer.

You are right, the word shifting was inappropriate. I have revised it as follows.

Revised: page, line,

　Page 7, Line 99-103

The purpose of this study is to examine the variables that influence the progressing stages of sarcopenia that impact robust, pre-sarcopenia, and sarcopenia in the residents of Oki-island. We hypothesized that depression and dementia have a positive effect, while hypertension has a negative effect, on the progression stages of sarcopenia.

　Page 21, Line 315-318

We found multiple factors associated with the progression from robust to pre-sarcopenia and sarcopenia. In the progression of stages from robust to pre-sarcopenia, in addition to age, depression and hypertension were significantly associated with the participants’ status.

2. SARC-F is not required for sarcopenia diagnosis using AWGS 2019, but can be diagnosed by grip strength, walking speed, and SMI. Therefore, the authors should address a clear reason for using AWGS2014.

**Reply:**

As you pointed out, the SARC-F is not required for picking up sarcopenia because this study measured all participants. However, in this study, the speed of 5m walking was measured, so the speed of 6m walking is unknown. 6m walking speed should be measured by AWGS 2019 standards. Hence, we could not classify them according to the AWGS2019 criteria. The sentence about SARC-F has been removed from Limitation. I appreciate your pointing out our error.

**Revised:** page 25, Line 374-377

Third, this study was conducted in 2016, and the speed of the 5 m gait was measured according to the AWGS 2014 criteria and not the AWGS 2019 criteria using the 6 m gait. Our future task is to a conduct a sarcopenia study using the AWGS 2019 criteria.

3. The comparison of the three groups cannot be mentioned by only the Kruskal-Wallis test (p15, 243-248 line). Multiple comparison analysis is required to confirm these results.
**Reply:**

In this part, we showed the trend of the three groups' characteristics. Then, we performed multivariate ordered logit regression analysis including the related factors and identified the significantly related factors (Table 2). Since the aim was achieved, we did not perform that multiple comparison analysis.

Minor comments:
1. In Table 1, the interquartile range should be indicated by the 1st quartile – 3rd quartile. In addition, the authors should add the items such as pre-sarcopenia, sarcopenia etc in the column.
**Reply:**

I rewrote Table1 as you instructed. I had not written the items, robust, pre-sarcopenia, and sarcopenia; thus, I added them. Thank you for pointing this out.

2. I could not find the S1 and S2 tables in this revised version.

**Reply:**

In accordance with the PLOS one MANUSCRIPT BODY FORMATTING GUIDELINES, the S1 and S2 tables (supporting information) were uploaded in a separate file and were not included in the manuscript.

3. The median of age was 74.9 years in this manuscript. However, the mean age of the first manuscript was also 74.9 years. Is it correct?

**Reply:**

I made an error: mean age was exactly 74.89 years old and median age was 75.0 years old. I have corrected the text.
